# Adaptive vehicle extraction in real-time traffic video monitoring based on the fusion of multi-objective particle swarm optimization algorithm

**DOI:** 10.1186/s13640-018-0381-8

**Published:** 2018-12-17

**Authors:** Shijun Yu, Shejun Deng

**Affiliations:** College of Civil Science and Engineering, Yangzhou University, No. 198 Huayang Road, Yangzhou, 225127 Jiangsu China

**Keywords:** Vehicle detection, Multi-objective particle swarm optimization algorithm, Real-time traffic video, Adaptive vehicle extraction

## Abstract

In view of the problems in the real-time traffic video monitoring that the adaptive vehicle extraction is greatly affected by the environmental factors such as the illumination, noise, and so on; the missed detection and error detection rate is high; and it is difficult to meet the robustness and the real-time performance at the same time, a kind of method for the adaptive vehicle extraction in real-time traffic video monitoring based on the fusion of multi-objective particle swarm optimization algorithm is put forward. In this method, based on the multi-objective particle swarm optimization algorithm, adaptive binarization processing is carried out on the image first, and the interference points are removed by filtration through the erosion and expansion method. Simple and effective method is used to carry out the merger of the shadow line and the extraction of the real-time traffic video. In the algorithm, the information entropy in the target area and the symmetry characteristics of the vehicle tail are used to screen and identify the region of interest, which has reduced the missed detection and error detection rate of the algorithm. The multi-objective particle swarm optimization algorithm is used to extract the vehicle boundaries and has achieved relatively good effect. The results show that the detection accuracy is 89% and the average operating speed is 17.6 frames/s during the processing of the real-time traffic video with the resolution of 640 × 480.

## Introduction

Statistics show that the direct economic loss caused by vehicle traffic accidents per minute in the world is as high as US$23 billion,[1, 2] and vehicle rear end collisions account for the largest proportion in the traffic accidents. In order to reduce the incident of traffic accidents, research and development of the intelligent vehicle assistant driving system has been carried out, in which the real-time traffic video vehicle detection technology is the core aspect of the system. It is one of the core technologies in the intelligent transportation system.

In [3], the authors proposed a coordinator traffic diffusion (CTD) method for redirecting excess traffic from the coordinator to the receiver in an electrocardiogram (ECG) medical application. The CTD router that implements the CTD design automatically redirects ECG data traffic to the aggregation node without involving the coordinator, so it can accurately transmit reliable real-time ECG monitoring services. The CTD design passes through the TI CC2530 Zigbee platform and NS2 simulation test. The experimental results show that the CTD design can help the router successfully and successfully transmit real-time ECG data samples at the optimal transmission rate of 24 kb/s. The original Zigbee design could not achieve this performance. In the literature [4], the author proposes a *k*-means grid density clustering algorithm based on artificial immune network, which divides the vehicle data into appropriate clusters and marks the density for monitoring and analyzing traffic conditions. Simulation results show that the algorithm has higher efficiency and stability than traditional methods. In the literature [5], the author proposes a new method to process geospatial data streams on grid computing resources. This is accomplished by submitting a continuous processing job to the grid that continuously polls the sensor data repository using the relevant open GIS standard. To evaluate this approach, a road traffic monitoring application was developed to process GPS observation streams from the fleet. Specifically, a Bayesian map matching algorithm is executed that matches each GPS observation with a link on the road network. The results show that more than 90% of the observations are correctly matched, and the method used can achieve timely results of performing linear time geoprocessing operations every 60 s. A number of recommendations have been made for grid job scheduling mechanisms, shortcomings in the OGC Web Processing Service Specification, and IO bottlenecks in OGC data services. The machine vision technology is used to detect and track the vehicles in front, which has the advantages of large volume of information, low cost, good robustness, and so on.[6–8]

In this field, a lot of technologies have been put forward, such as a kind of detection method based on the integration of the binocular stereo vision. Although the positioning is relatively accurate, the calculation volume is huge, and special hardware support is required. A method for obtaining the road surface gray threshold is implemented. However, it is impossible to solve the problem of the gray changes in the pavement. For example, in [9], the authors developed a method for remotely monitoring bridge deflection in real time. From a practical point of view, the main feature of the method is the ability to make free reference estimates for bridge deflection. For this purpose, the formulation uses the measured rotation and curvature of the bridge. The feasibility of this method was tested on two different highway bridges. Both bridges are equipped with many different fiber optic sensors, including inclinometers and strain gauges. Conventional transducers and displacement measuring devices were also used in parallel to evaluate the performance of the proposed method. A series of load tests were performed at two bridge sites, including the use of trucks of known weight; in [10], the author developed a simple and accurate analysis model for predicting the queuing delay of real-time multimedia service flows in WLANs based on non-isomorphic random access. This results in an easy analysis to meet the queuing delay specification for many streams. In particular, the results address the feasibility of ensuring the average delay required for a set of User Datagram Protocol (UDP) streams supporting real-time multimedia services in a WLAN. Based on the model feasibility analysis, the authors further developed an optimization technique to minimize the delay of traffic flow. In addition, they also proposed a decentralized algorithm and reported its implementation, and presented a wide range of simulation and experimental tracking-based results to demonstrate the accuracy of the model and the performance of the algorithm.

The disadvantage of using the optical flow method to identify the location of obstacles is that the static targets cannot be detected.[11, 12] In a vehicle detection method, the extraction and matching of the feature symbols is not required. However, this method requires the acquisition of the prior knowledge in the traffic scene in advance. For example, in the literature [13], the author proposes a new adaptive energy management strategy with better fuel efficiency than the original and traditional dynamic programming-based control strategies; in [14], the author introduces a vehicle-based light detection and A stepwise method of extracting transmission lines and towers from ranging (lidar) data was evaluated by two data sets obtained using the RIEGL VMX-450 system. The average integrity, correctness, and quality of the power lines extracted on the two data sets were 0.92, 0.99, and 0.91, respectively, and the positional accuracy including the root mean square error and the maximum error were 0.07 and 0.0502 m, respectively. The results show that the method extracts transmission lines with good thematic and positional accuracy from large-scale on-board lidar data. There is a method based on the integration of the knowledge, and the insufficiency of this method is that the error detection rate in the complex environment will increase significantly. The GOLD and RALPH systems make use of the reconstructed image to extract the road information. However, the computation is excessively large. In addition, parallel SIMD hardware structure needs to be designed and operated. In China, the vehicle detection method based on the fusion of the wavelet transform is applied. However, it fails to meet the wide adaptability of the system matching [15, 16].

In order to solve the above problems, a kind of method for the adaptive vehicle extraction in the real-time traffic video monitoring based on the fusion of vision is put forward in this paper. In this algorithm, the optimal threshold value is extracted through the method of local and dual between-class variance synthesis to extract the shadow at the bottom of the vehicle more effectively. The morphological method, the function symmetry, and the information entropy principles are adopted to carry out multi-objective analysis and discrimination on the vehicles in front, which has improved the robustness of the system significantly and reduced the missed detection and error detection rate. In the process of the real-time performance optimization, the computational flow of the target particle swarm optimization algorithm is improved in this paper, so that the real-time performance of the system can meet the requirements for the autonomous driving of the intelligent vehicles.

## Real-time traffic video extraction

The binarization processing aims to obtain the shadow area at the bottom of the vehicle. How to extract this area more effectively has always been an important issue in the mode detection section.

Firstly, the grayscale processing is carried out on the image and the empirical formula is adopted as follows:

1 $$ R=G=B=0.299R+0.587G+0.144B $$

The maximum between-class variance (OTSU) method is widely used in the process of the pattern detection, which can adaptively select the threshold value [10] and distinguish between the background and the target area. Firstly, the characteristic parameters of the grayscale image need to be calculated as follows:

2 $$ \mu \kern0.5em =\kern0.5em {\omega}_0{\mu}_0\kern0.5em +\kern0.5em {\omega}_1{\mu}_1 $$

3 $$ {\sigma}^2(K)={\omega}_0{\left({\mu}_0\hbox{-} \mu \right)}^2+{\omega}_1{\left({\mu}_1\hbox{-} \mu \right)}^2 $$

In the equation, *ω*_0_ and*ω*_1_ stand for the probability of the appearance of the gray value of the pixel dots in the background and the target area, respectively, *μ*_0_ and *μ*_1_ stand for the mean gray value of the pixel dots in the background and target area, respectively, *μ*stand for the mean statistical value of the overall image grayscale, and *σ*^2^(*K*) stand for the between-class variance of the background area and the target area, in which *K* is changed among 1, 2, ..., *m*. The value for *K* when the variance is maximum is obtained, that is, the value for K at max *σ*^2^( *K*) is the optimal threshold value.

**Fig. 1:**
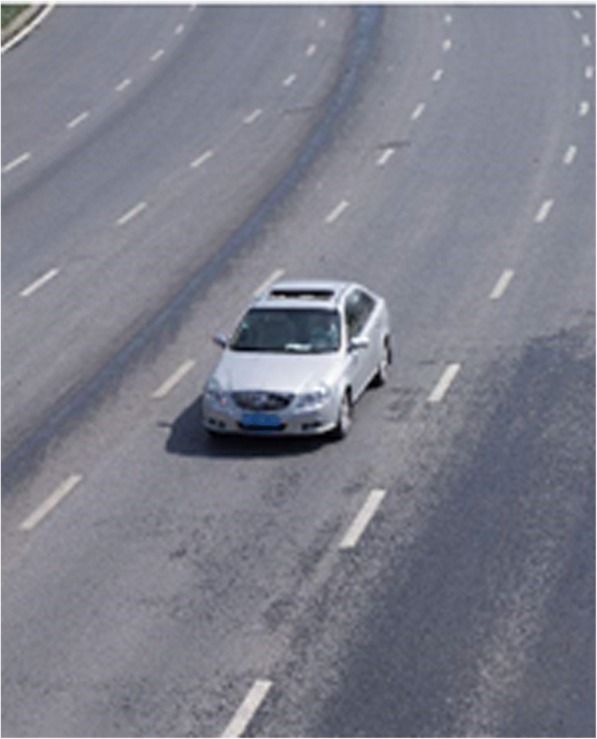
Original image

**Fig. 2:**
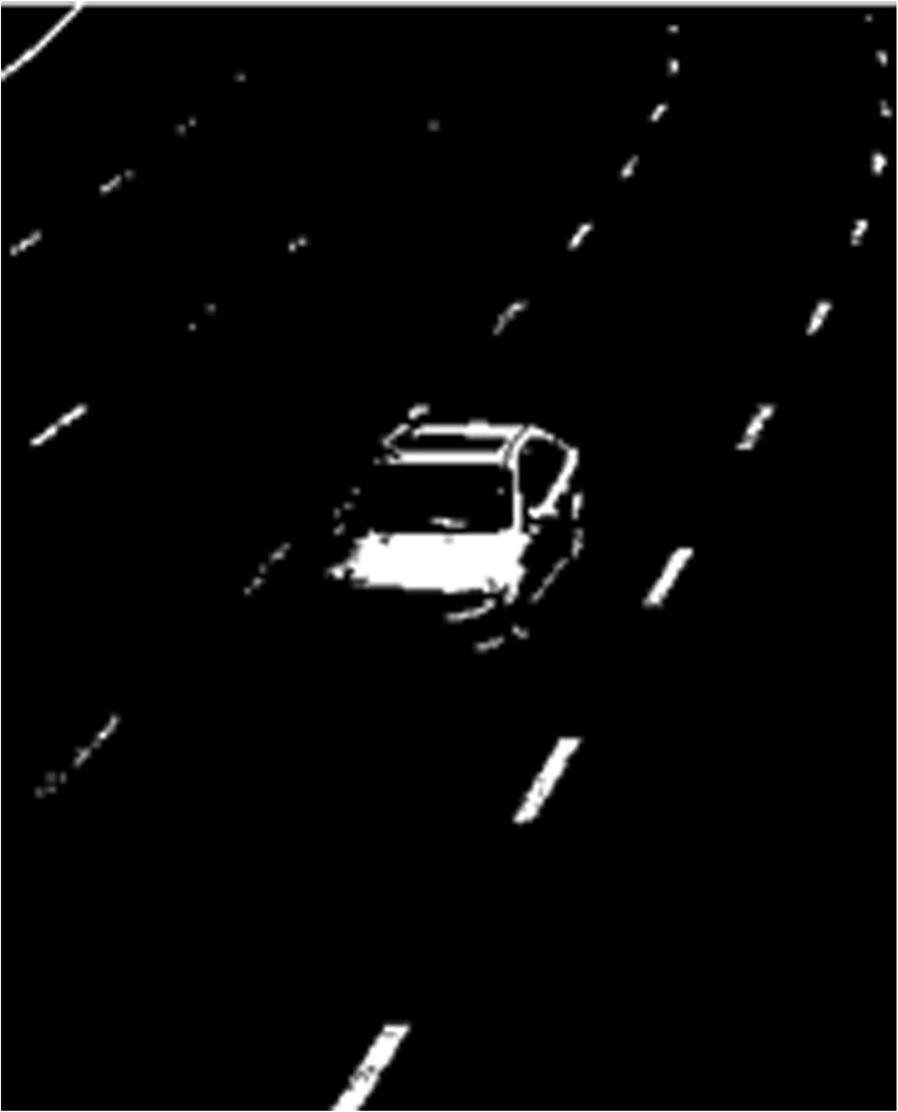
Single OTSU segmentation

It is impossible to separate the road pavement pixels and the shadow at the bottom of the vehicle accurately by the application of single OTSU method, as shown in Figs. 1 and 2 as the following.

**Fig. 3:**
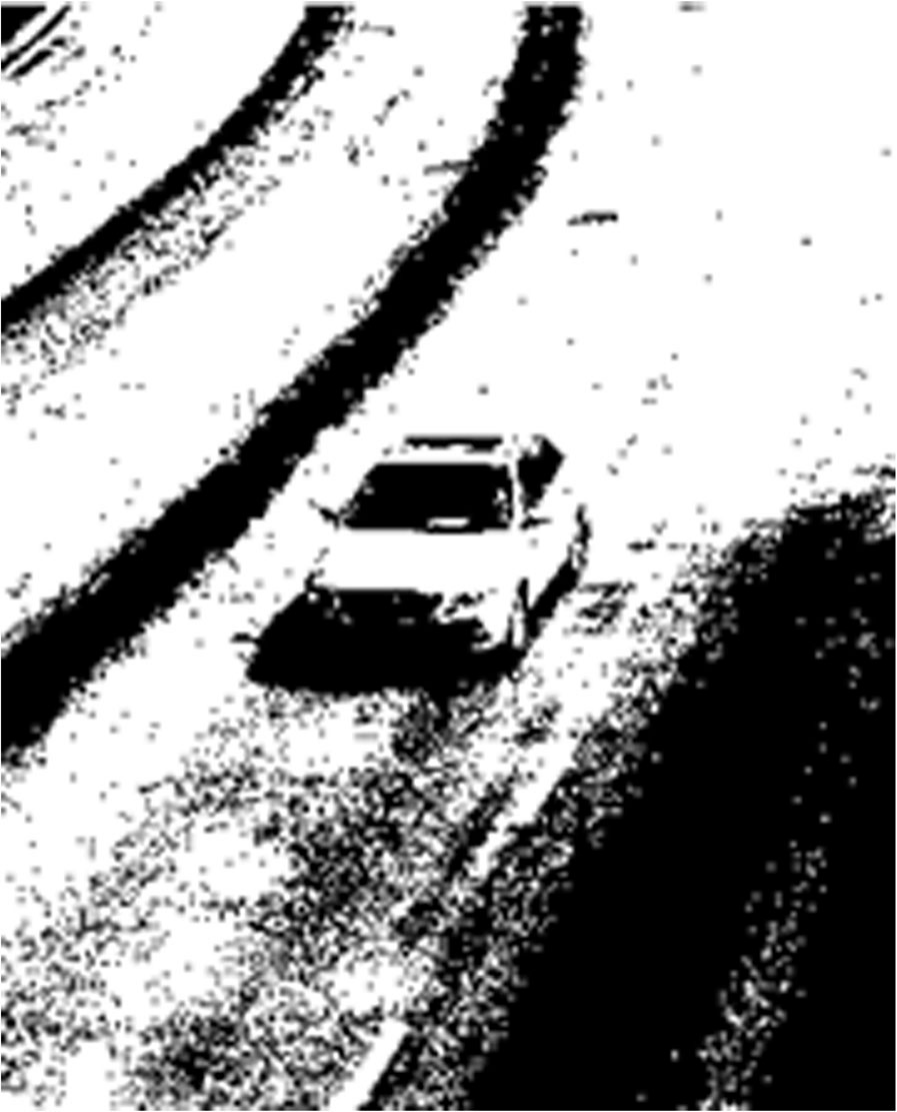
Dual OTSU segmentation

The results obtained from the processing cannot meet the system requirements. Therefore, the dual OTSU method is put forward to carry out image segmentation. And the method is as follows:

1. Firstly, the OTSU is used to calculate the overall image threshold value *T*_1_.
2. All the pixel dots in the graph are traversed and the threshold value *T*_1_ is used to carry out classification. Those greater than *T*_1_ are classified as the background.
3. For the target pixel that remains after the screening in (2), the OTSU method is used again to obtain the new threshold value *T*_2_.
4. *T*_2_ is adopted as the segmentation threshold value to carry out the binarization on the image again. Those greater than *T*_2_ are classified as the background, the pixel value is set to 255. Those smaller than *T*_2_ are as the target pixels, and the pixel value is set to 0. The results can be obtained after the processing of the dual OTSU method, as shown in Fig. 3 as the following.

Through the processing of multiple images, it has been found that some shadow areas obtained sometimes show the gap or adherence to the surrounding environment and other situations. In view of the above situations, morphological methods such as dilation and corrosion and so on can be used for processing.

**Fig. 4:**
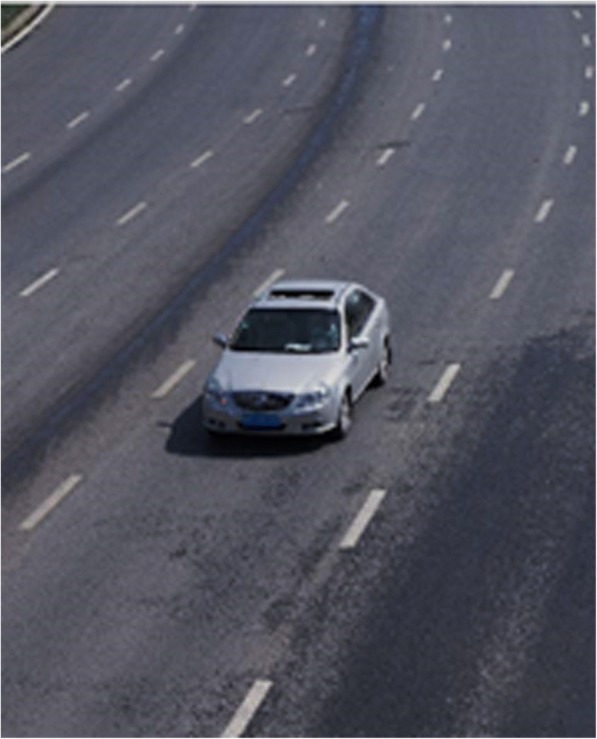
Corrosion treatment

**Fig. 5:**
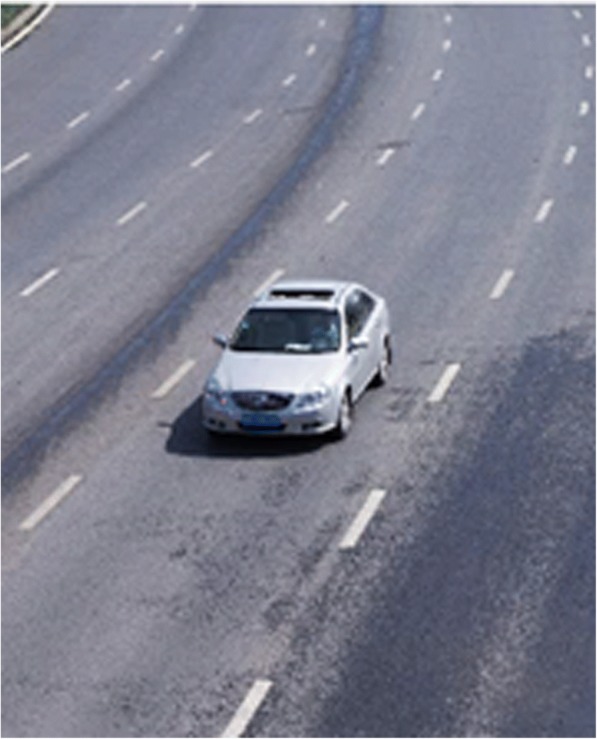
Expansion treatment

Corrosion and dilation processing is carried out on the binary image segmented previously. The processed image is filtered to remove the impurities and noises in the original image. In addition, it has relatively good shape features, as shown in Figs. 4 and 5.

After the above-mentioned processing, although the shadow area at the bottom of the vehicle with relatively good properties has been obtained, there was still certain interference present. Firstly, the starting and ending positions of the shadow line is searched row by row from top to bottom and from left to right, so as to determine the length and position of the shadow line. When the Eq. (4) is met, it is considered that the starting point *x*_start_ of the shadow line has been found; and when the Eq. (5) is met, it is considered that the ending point *x*_end_ of the shadow line has been found as follows:

4 $$ \left\{\begin{array}{l}f\left(x-1,y\right)-f\left(x,y\right)=255\\ {}f\left(x,y\right)-f\left(x+1,y\right)=0\end{array}\right. $$

5 $$ \left\{\begin{array}{l}f\left(x,y\right)-f\left(x-1,y\right)=0\\ {}f\left(x+1,y\right)-f\left(x,y\right)=255\end{array}\right. $$

There are a number of line segments with different lengths in the image, while the length of the shadow line at the bottom of the vehicle in the image should be within a certain range. In accordance with the calibration of the camera position and the parameters, this range will be changed with the different rows that the shadow line is located. On the basis of this feature, a threshold value is selected for each row in the system. If the length of the detected shadow line *length = x*_end_ *− x*_start_ is excessively different from the threshold value, the inference of the shadow line can be filtered out. And the corresponding relation expression is as follows:

6 $$ w\approx \frac{w_p}{H}\left(y-\frac{h}{2}\right) $$

In the equation, *w* stands for the length scalar of the shadow line in the image, that is, pixel; *w*_p_ stands for the actual width of the vehicle, that is, m; *H* stands for the height of the optical axis of the camera from the ground, for which the value of 1.6 m is taken; *y* stands for the row number of the target in the direction of the longitudinal axis of the image, that is, pixel; and *h* stands for the height of the image, that is, pixel. When the Eq. (5) is met, the shadow line can be considered as the shadow at the bottom of the vehicle as the following.

7 $$ 0.75w<\mathrm{length}\kern0.5em =\kern0.5em {x}_{\mathrm{start}}\kern0.5em -\kern0.5em {x}_{\mathrm{end}}\kern0.5em <\kern0.5em 1.25w $$

On the basis of the multi-objective particle swarm optimization algorithm for the extraction of the lane line, it is possible to filter out the shadow lines that are outside the lane line and have no intersection with the lane line. At the same time, in order to extract the suitable shadow area at the bottom of the vehicle, the adjacent shadow lines in the *y* axis direction are merged and traversed row by row to obtain the shadow straight line. When|*y*_1_ − *y*_2_| < *T* is met, the two rows are merged, and four pixels are taken as the value for *T*.

The position parameters of the real-time traffic video obtained by the above steps may exceed the boundary of the image. Therefore, it is necessary to make adjustment to the real-time traffic video in accordance with the boundary information of the image to prevent the program from giving an error alert. It is assumed that *W* = 640, *H* = 480, and the adjustment method is as follows:

8 $$ \left\{\begin{array}{l} If\ {R}_{v\_x}<0,\mathrm{then}\ {R}_{v\_x}=0\\ {} If\ {R}_{v\_y}<0,\mathrm{then}\ {R}_{v\_y}=0\\ {} If\ {R}_{v\_\mathrm{width}}<0,\mathrm{then}\ {R}_{v\_\mathrm{width}}=0\\ {} If\ {R}_{v\_x}+{R}_{v\_\mathrm{width}}>W, then\ {R}_{v\_\mathrm{width}}=W-{R}_{v\_x}\\ {} If\ {R}_{v\_y}+{R}_{v\_\mathrm{height}}>W, then\ {R}_{v\_\mathrm{height}}=W-{R}_{v\_y}\end{array}\right. $$

**Fig. 6:**
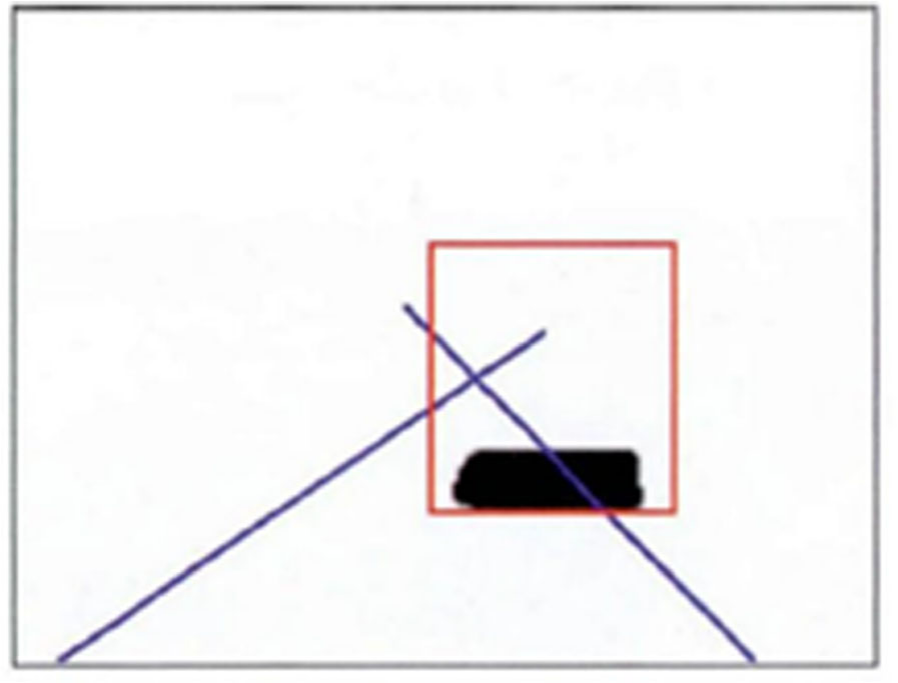
Extraction of the real-time traffic video

The real-time traffic video extracted is shown in Fig. 6 as the following.

## Multi-objective particle swarm optimization method

In the road detection, the texture features are used to represent the attributes of the structure and surface of objects. Within the candidate area that contains the vehicle, the jump of the grayscale value may occur at the edge of the vehicle. At the same time, feature pixel modules such as the automobile headlights, license plates, bumpers, and so on are included in the vehicle rear part. It can be seen that in the real-time traffic video for the rear of the vehicle, rich texture information should be available. For a grayscale image *I*, pi stands for the probability of the occurrence of a point where the gray value is *i*. Then the expression of the entropy value is as follows:

9 $$ H(I)=-\sum \limits_{i=0}^{255}{p}_i{\log}_2{p}_i $$

The entropy value of the image will increase significantly in the candidate area that contains the vehicle. For the road pavement area, since the gray value of the road pavement is relatively single, the pixel information value included is relatively low. Hence, this property can be used to filter out some areas with relatively small entropy value. It is assumed that *j* stands for the row number in the real-time traffic video with the height *h* that is extracted above, *H* (*j*) stands for the entropy value of each row, then the mean value of the information entropy in the real time traffic video is as follows:

10 $$ \overline{H}(I)=\frac{1}{h}\sum \limits_{j=1}^hH(j) $$

**Fig. 7:**
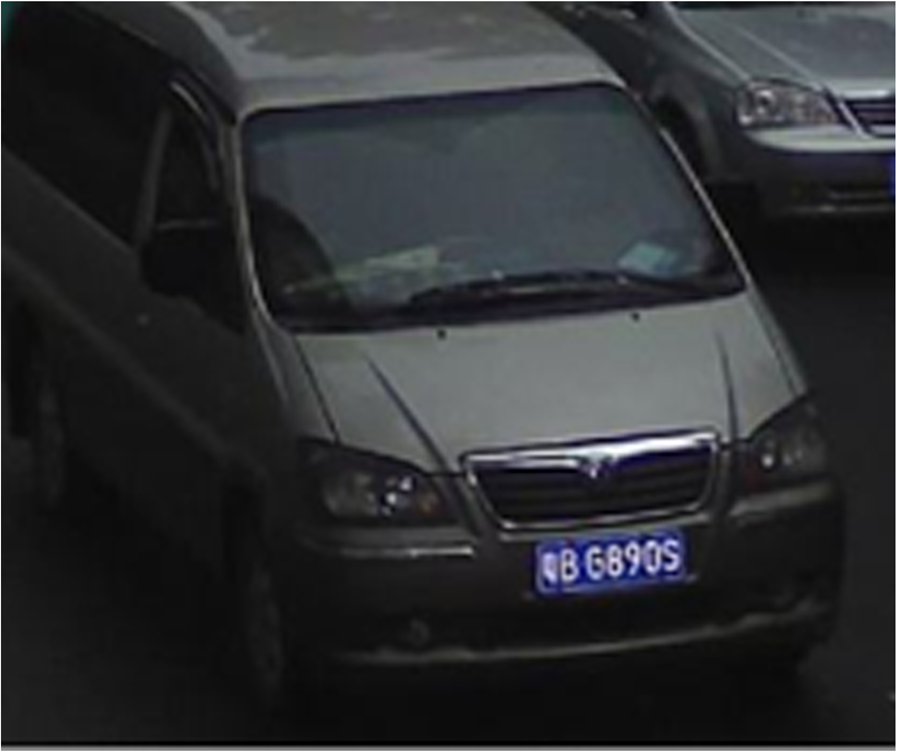
Vehicle area

**Fig. 8:**
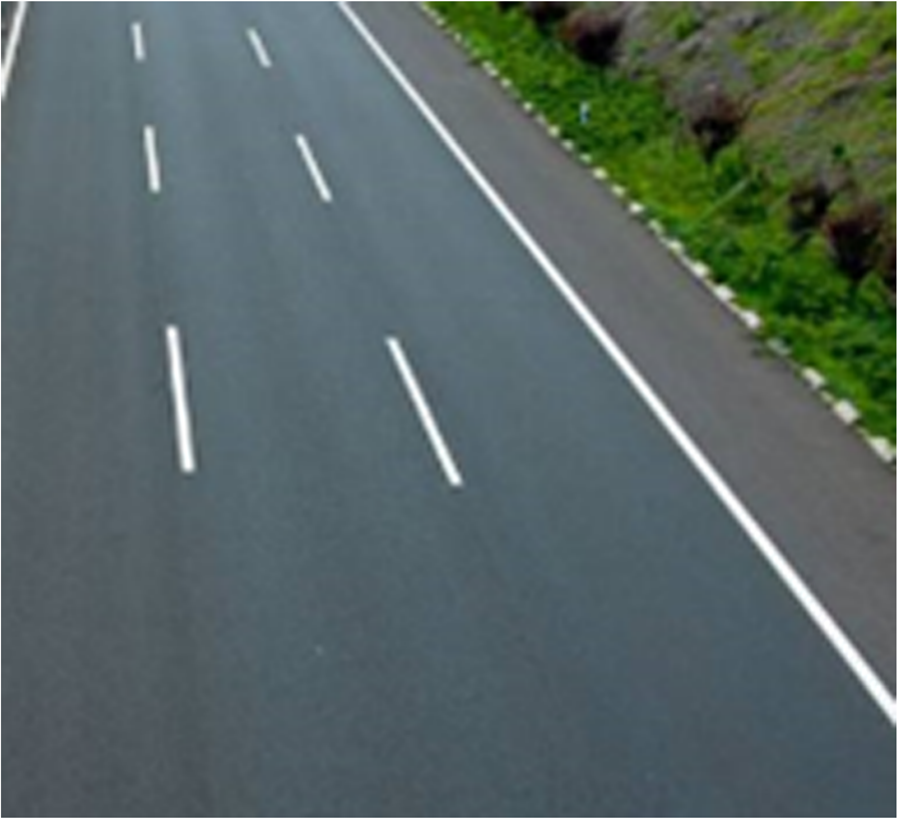
Road area

Figure 7 shows the vehicle area with the information entropy of 4.8; Fig. 8 shows the road area with the entropy value of 2.2. In the experiment, 80 different images are selected for processing. The final threshold value is selected as *T*_E_ = 3.1. Hence, when $\overline{H}(a)>{T}_E$, it can be considered that the real-time traffic video contains the vehicle information; otherwise, the real-time traffic video can be filtered out.

The mathematics principle is applied. It is assumed that *R* (*x*) stands for the linear continuous function in the real-time traffic video. Then it can be split into the odd function *R*_*o*_ (*x*) and the even function *R*_*e*_ (*x*). Hence, the symmetry of a function can be determined in accordance with the proportion of the odd and even functions separated.

For the above real-time traffic footage extracted, it is assumed that the size of the area is w × h, and the axis of symmetry is ${x}_s=\frac{w}{2}$, then for the *y*th row line segment in the image, the expressions for the even function and the odd function are as follows:

11 $$ {R}_e\left(u,y\right)=\frac{1}{2}\left[R\left({x}_s+u,y\right)+R\left({x}_s-u,y\right)\right] $$

12 $$ {R}_o\left(u,y\right)=\frac{1}{2}\left[R\left({x}_s+u,y\right)-R\left({x}_s-u,y\right)\right] $$

It is necessary to carry out correction on the even function in the algorithm so that the corrected mean value is the same as the odd function and approaches zero. Hence, the energy function can be used to compare the relationship between the two. After the correction, the following can be obtained

13 $$ {R}_e^{\hbox{'}}\left(u,y\right)={R}_e\left(\mathrm{u},y\right)-\frac{1}{w}\sum \limits_{v=1}^w{R}_e\left(v,y\right) $$

Thus, the energy functions of the odd function and the even function can be obtained as the following

14 $$ E\left[{R}_o\left(u,y\right)\right]=\sum \limits_{u=-w/2}^{w/2}{R}_o^2\left(u,y\right) $$

15 $$ E\left[{R}_e^{\hbox{'}}\left(u,y\right)\right]=\sum \limits_{u=-w/2}^{w/2}{\left[{R}_e\left(u,y\right)-\frac{1}{w}\sum \limits_{v=o}^w{\mathrm{R}}_e\Big(v,y\Big)\right]}^2 $$

Therefore, the symmetry of the pixel in the *y*th row can be measured and calculated as the following

16 $$ S\left[R\left(u,y\right)\right]=\frac{E\left[{R}_e^{\hbox{'}}\left(u,y\right)\right]-E\left[{R}_o\left(u,y\right)\right]}{E\left[{R}_e^{\hbox{'}}\left(u,y\right)\right]+E\left[{R}_o\left(u,y\right)\right]} $$

And the following can be obtained

$$ \left\{\begin{array}{l}\mathrm{Fully}\ \mathrm{symmetry}\kern2.05em S\left[I(x.y)\right]=1\\ {}\mathrm{Asymmetry}\kern3.6em S\left[I\left(x,y\right)\right]=0\\ {}\mathrm{Inverse}\ \mathrm{symmetry}\kern1em S\left[I\left(x,y\right)\right]=-1\end{array}\right. $$

**Fig. 9:**
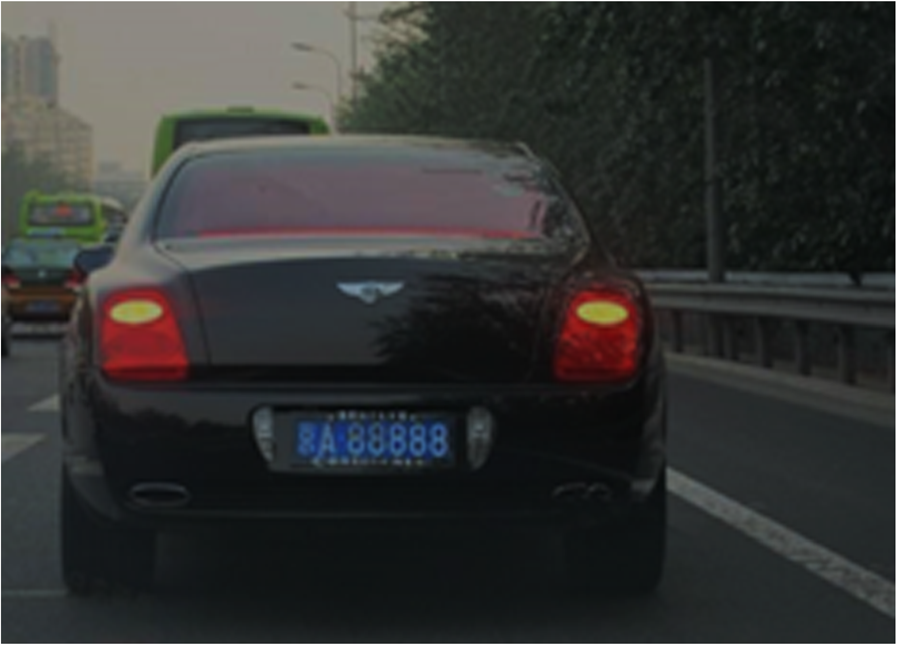
Vehicle area

**Fig. 10:**
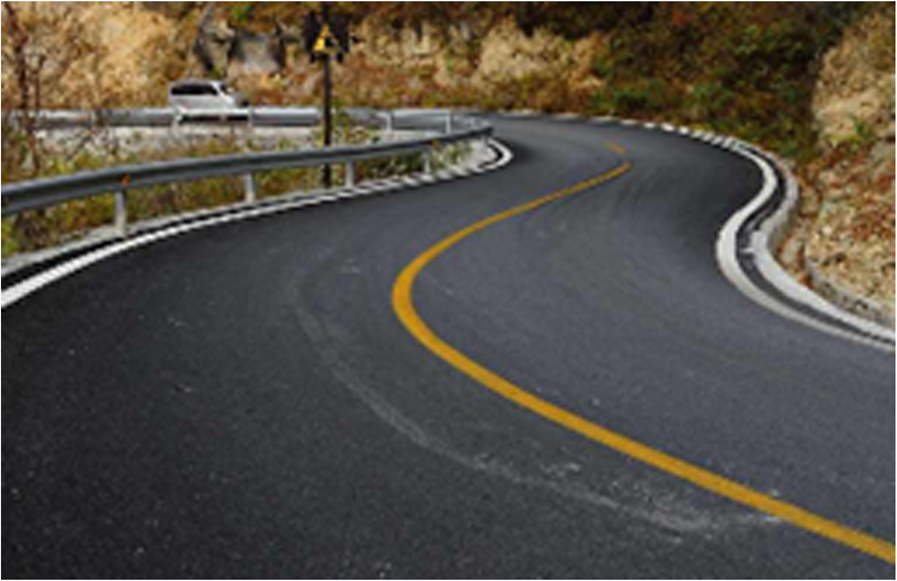
Background area

The symmetry measure in Fig. 9 is *S* = 0.304, and in Fig. 10, *S* = 0.031. After the statistics on a large number of images, the threshold value is finally selected as *T*_s_ = 0.15. When *S* > 0.15, it can be determined as the real-time traffic video of the vehicle; otherwise, the area is deleted.

## Adaptive vehicle extraction in the real-time traffic video monitoring

After the above processing steps are completed, relatively accurate real-time traffic video vehicle area can be obtained. In this area, the target particle swarm optimization algorithm universal template operator [11] is adopted to carry out the edge detection and obtain the gradient value. The eight templates in Fig. 12 are used to carry out detection on the points in the area, respectively. Among them, the maximum output value and its direction are taken as the grayscale value and the direction obtained after the point is processed.

In the next session, example is given to illustrate the use of the target particle swarm optimization algorithm to detect the pixels in the real-time traffic video. It can be seen that the computation method for any pixel dot and the *M*_1_ template is as follows:

17 $$ {e}_0={p}_0+2\times {p}_1+{p}_2-{p}_4-2\times {p}_5-{p}_6 $$

For any pixel dot, it is required that eight operations similar to the above-mentioned operation should be completed to obtain the accurate results. Hence, it can be seen that the detection of a point requires 16 multiplication operations and 40 addition and subtraction operations, which will slow down the operating speed of the system. To solve this problem, an improved method is put forward. And the variables are introduced in accordance with the method as the following.

18 $$ \left\{\begin{array}{l}{x}_0={p}_0-{p}_4\\ {}{x}_1={p}_1-{p}_5\\ {}{x}_2={p}_2-{p}_6\\ {}{x}_3={p}_3-{p}_7\end{array}\right.,\kern0.5em \left\{\begin{array}{l}{y}_0={x}_0+{x}_1\\ {}{\mathrm{y}}_1={x}_1+{x}_2\\ {}{y}_2={x}_2+{x}_3\\ {}{y}_3={x}_3-{x}_0\end{array}\right. $$

The speed of the operation is optimized, and the following result can be obtained

19 $$ \left\{\begin{array}{l}{e}_0={y}_0+{y}_1\\ {}{e}_1={y}_1+{y}_2\\ {}{e}_2={y}_2+{y}_3\\ {}{e}_3={y}_3-{y}_0\end{array}\right.,\left\{\begin{array}{l}{e}_4=-{e}_0\\ {}{e}_5=-{e}_1\\ {}{e}_6=-{e}_2\\ {}{e}_7=-{e}_3\end{array}\right. $$

**Fig. 11:**
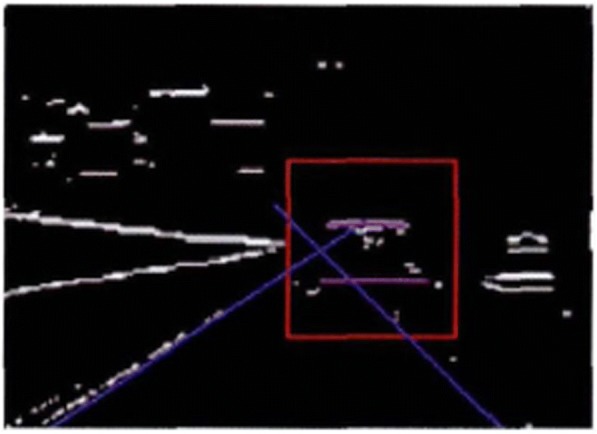
Horizontal boundary extraction

**Fig. 12:**
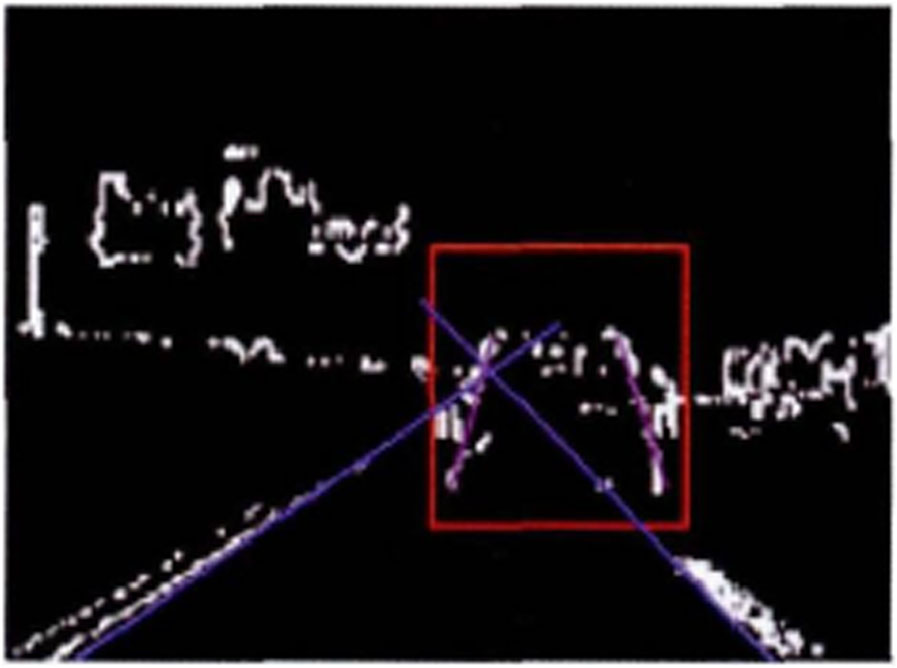
Vertical boundary extraction

The multi-objective particle swarm optimization algorithm is a relatively common and mature method for the extraction of a straight line. The disadvantage is that the amount of computation is relatively huge. At this point, the multi-objective particle swarm optimization algorithm is applied only in the real-time traffic video, which has reduced the amount of calculation to a great extent. In this paper, limit is set at the angle of the straight line so as to further speed up the calculation process. In the process of extracting the horizontal edge, let −5^°^ < *θ* < 5^°^. And in the process of extracting the longitudinal edge, let 60^°^ < *θ* < 120^°^. The results are shown in Figs. 11 and 12 as the following.

It is assumed that the vertical left (right) boundary line is subject to the leftmost (right) point and the slope value tends to the infinity, then the outer boundary of the rectangular vehicle can be obtained.

## Verification results and discussions

**Fig. 13:**
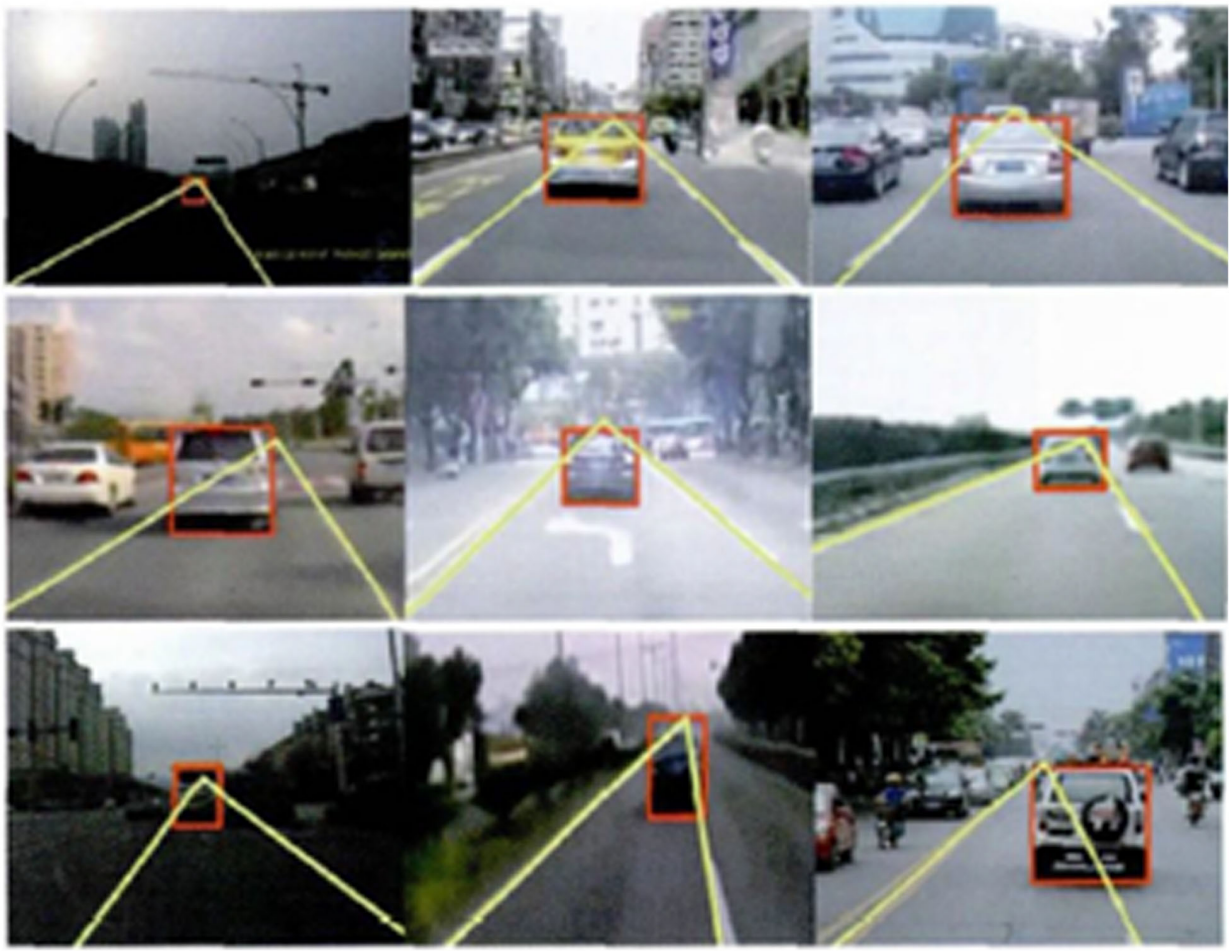
Processing results under different road conditions and environments

All the algorithms involved in this paper are programmed in opencv and c++ language, which are configured and debugged in the opencv2.4.5 database under the vs2010 platform. They can run stably in Windows XP and Windows 7 operating systems. The processor of the computer adopts the Intel(R) Pentium(R) Dual-Core CPI T4200 2.0 GHz, with 2.00 GB memory. The CCD camera is installed in the rear central position of the front windshield of the vehicle. The captured real-time traffic video image is 640 × 480 structured road image. The urban road has the characteristics of poor road quality, complicated background, ever-changing environment, and so on, which have posed relatively high requirements for the robustness of the algorithm. In order to evaluate the effectiveness of the algorithm put forward in this paper, nearly a hundred images of the road in front are tested. These images were taken in different road environments by using different visual equipment. And they have different grayscale characteristics and interference noises, as shown in Fig. 13. The results show that only five images cannot meet the detection requirements. Therefore, it can be seen that the algorithm can achieve good results in a variety of environments. The real-time performance is an important indicator for the overall performance of a method. The size of the original image is 640 × 480. The pixel is 307,200. And the mean time for the processing of the image per frame is 56.81 ms.

**Fig. 14:**
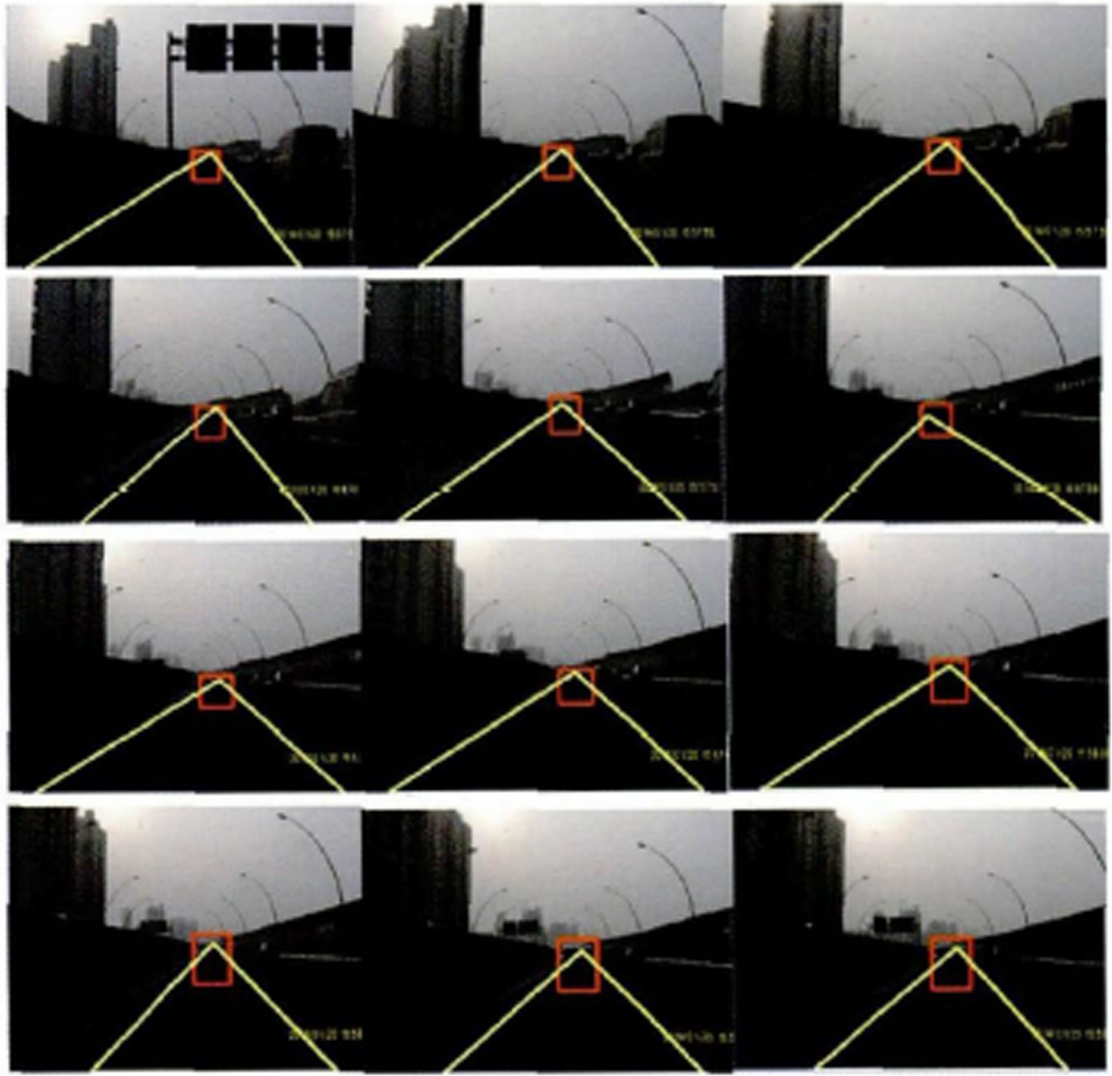
Is taken for the interval (17 frames)

The mean is 17.6 frames/s. If the driving speed of the intelligent vehicle is 40~80 km/h, the traveling speed of the vehicle is 11.1~22.2 m/s accordingly. When the vehicle speed reaches the upper limit, the algorithm will update the road information once every 1.26 m. Figure 14 shows the operation result of the real time traffic video processing during the vehicle operation. The real-time performance of this algorithm can meet the operation requirement of 15 frames/s relatively well, which has provided more sufficient theoretical basis for the design of the sensor fusion algorithm in the future.

## Conclusions

In this paper, a kind of detection method for the moving vehicles in front based on the intelligent vehicle and structured roads is put forward. In the process of binarization, the method of combining the local statistics and the dual maximum between-class method is adopted to select the optimal threshold value and make the algorithm more inclusive to the images with different parameters, thereby enhancing the robustness of the system. Relatively good effect has been achieved in the corrosion and expansion processing, which has enhanced the shape features of the shadow area without imposing pressure on the operating speed at the same time. The extraction of the shadow area and the real-time traffic video is improved on the basis of the traditional algorithm, and the line segments with the length not meeting the requirements are filtered out. In the link of the multi-objective particle swarm optimization, the interference of the road areas and the background areas that may be selected as the real-time traffic video is deleted through the methods such as the information entropy, the symmetry research, and so on, which has reduced the error detection rate of the system. The directional template of the target particle swarm optimization algorithm is suitable for the design of this paper. In addition, the speed of the improved algorithm has been increased significantly. Finally, the horizontal and vertical boundaries of the vehicle are extracted through the multi-objective particle swarm optimization algorithm under the angle constraint conditions. The experiments show that both the robustness and the real-time performance can meet the expected requirements, which has laid a solid foundation for the multi-sensor fusion in the later stage.

## Abbreviation

MICV: Maximum inter class variance

## References

[1] Minardo A, Porcaro G, Giannetta D, Bernini R, Zeni L (2013). Real time monitoring of railway traffic using slope-assisted brillouin distributed sensors. Appl. Opt..

[2] Janecek A, Valerio D, Hummel KA, Ricciato F, Hlavacs H (2015). The cellular network as a sensor: from mobile phone data to real time road traffic monitoring. IEEE Trans. Intell. Transp. Syst..

[3] Tseng C (2013). Coordinator traffic diffusion for data-intensive zigbee transmission in real time electrocardiography monitoring. IEEE Trans. Biomed. Eng..

[4] Chen CM, Pi D, Fang Z (2013). Artificial immune k-means grid-density clustering algorithm for real time monitoring and analysis of urban traffic. Electron. Lett..

[5] Mccullough A, James P, Barr S (2012). Near real time geoprocessing on the grid: a scalable approach to road traffic monitoring. Int. J. Geogr. Inf. Sci..

[6] D'Andrea E, Ducange P, Lazzerini B, Marcelloni F (2015). Real time detection of traffic from twitter stream analysis. IEEE Trans. Intell. Transp. Syst..

[7] Chen L, Zhu D, Tian J, Liu J (2016). Dust particle detection in traffic surveillance video using motion singularity analysis. Dig Sig Proc.

[8] Helmi K, Taylor T, Zarafshan A, Ansari F (2015). Reference free method for real time monitoring of bridge deflections. Eng. Struct..

[9] Hoh B, Iwuchukwu T, Jacobson Q, Work D, Bayen AM, Herring R (2012). Enhancing privacy and accuracy in probe vehicle-based traffic monitoring via virtual trip lines. IEEE Trans. Mob. Comput..

[10] Gao Y, Tan CW, Huang Y, Zeng Z, Kumar PR (2016). Characterization and optimization of delay guarantees for real time multimedia traffic flows in ieee 802.11 wlans. IEEE Trans. Mob. Comput..

[11] Chen BH, Huang SC (2015). Probabilistic neural networks based moving vehicles extraction algorithm for intelligent traffic surveillance systems. Inf. Sci..

[12] Wu BF, Juang JH (2012). Adaptive vehicle detector approach for complex environments. IEEE Trans. Intell. Transp. Syst..

[13] Zhang S, Xiong R (2015). Adaptive energy management of a plug-in hybrid electric vehicle based on driving pattern recognition and dynamic programming. Appl. Energy.

[14] Guan H, Yu Y, Li J, Ji Z, Zhang Q (2016). Extraction of power-transmission lines from vehicle-borne lidar data. Int. J. Remote Sens..

[15] Gong W, Yan X, Liu X, Cai Z (2015). Parameter extraction of different fuel cell models with transferred adaptive differential evolution. Energy.

[16] Fortin B, Lherbier R, Noyer JC (2012). Feature extraction in scanning laser range data using invariant parameters: application to vehicle detection. IEEE Trans. Veh. Technol..

